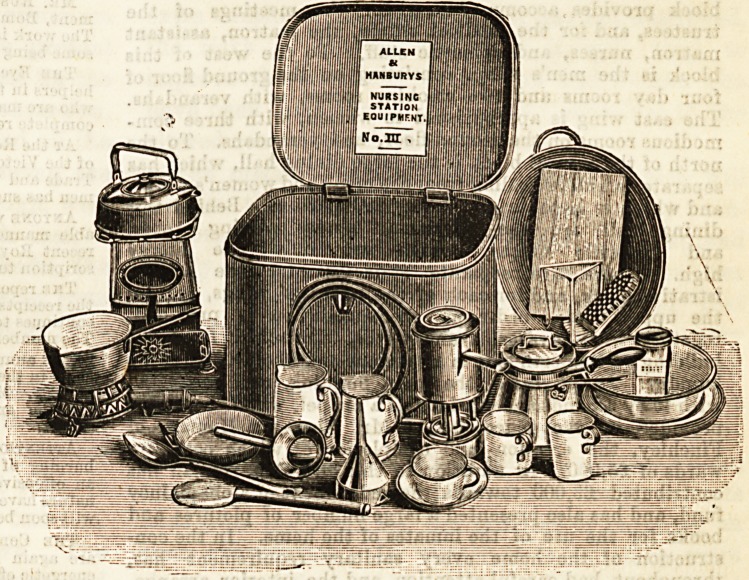# Ambulance Equipments

**Published:** 1893-08-19

**Authors:** 


### PRACTICAL DEPARTMENTS.

### AMBULANCE EQUIPMENTS.

**In a little handbook, dealing with the ambulance station necessaries, Messrs. Allen and Hanbury wisely urge the importance of providing a complete equipment from the beginning, wherever a nursing station is started, such a course proving over and over again to be the cheapest in the long run. For comparatively speaking a small sum an equipment, complete in itself, can be obtained, containing all the things needful for nursing, for the rendering of "first**

**aid," and for helping the poor in their own homes. A London guild has recently proved, in a most practical manner, the value of such an arrangement. During twelve months more than 500 cases were attended by the lady members, and the cost of providing the necessary appliances was originally some £10, with an additional £5 for replenishing during the year. A very full and complete list has been arranged by Dr. Tunstall, and the equipments can be inspected at either of Messrs. Allen and Hanbury's establishments. A very convenient portable arrangement in four boxes has also been prepared. Our illustrations gives a good idea of the contents of Box No. 3, which is fully supplied with cooking things.**

is a tin box, 16 inches long, and the contents are as follows: 1 gas or oil stove; 3 saucepans, one with jar; 1 strainer; 2 plates; 1 frying pan: 1 spirit lamp, with stand; 1 kettle; 1 funnel; 2 trays; 1 chopping board; 1 bath; 2 jugs; 1 scrubbing brush; 2 mugs, and cup and saucer; 2 large spoons (wood and tin); 2 basins. The price ranges from £1 15s. Boxes No. 1 and 2 contain "sanitary nursing necessaries," and "dressings, nourishments, &c." The latter is provided with a double folding lid, forming a table. The fourth box provides linen, for binding, and also for dressings. The whole of this most convenient and ample outfit can be had for a cost of £10 5s., and in most cases will be found to meet all needs which a district nurse or member of the association will have to face in the course of her ordinary work amongst the sick poor.

## Figures and Tables

**Figure f1:**